# Traumatic brain injury: pathophysiology for neurocritical care

**DOI:** 10.1186/s40560-016-0138-3

**Published:** 2016-04-27

**Authors:** Kosaku Kinoshita

**Affiliations:** Division of Emergency and Critical Care Medicine, Department of Acute Medicine, Nihon University School of Medicine, 30-1 Oyaguchi Kamimachi, Itabashi-ku, Tokyo, 173-8610 Japan

**Keywords:** Traumatic brain injury, Pathophysiology, Neurocritical care, Catecholamine, Hyperglycemia

## Abstract

Severe cases of traumatic brain injury (TBI) require neurocritical care, the goal being to stabilize hemodynamics and systemic oxygenation to prevent secondary brain injury. It is reported that approximately 45 % of dysoxygenation episodes during critical care have both extracranial and intracranial causes, such as intracranial hypertension and brain edema. For this reason, neurocritical care is incomplete if it only focuses on prevention of increased intracranial pressure (ICP) or decreased cerebral perfusion pressure (CPP). Arterial hypotension is a major risk factor for secondary brain injury, but hypertension with a loss of autoregulation response or excess hyperventilation to reduce ICP can also result in a critical condition in the brain and is associated with a poor outcome after TBI. Moreover, brain injury itself stimulates systemic inflammation, leading to increased permeability of the blood–brain barrier, exacerbated by secondary brain injury and resulting in increased ICP. Indeed, systemic inflammatory response syndrome after TBI reflects the extent of tissue damage at onset and predicts further tissue disruption, producing a worsening clinical condition and ultimately a poor outcome.

Elevation of blood catecholamine levels after severe brain damage has been reported to contribute to the regulation of the cytokine network, but this phenomenon is a systemic protective response against systemic insults. Catecholamines are directly involved in the regulation of cytokines, and elevated levels appear to influence the immune system during stress. Medical complications are the leading cause of late morbidity and mortality in many types of brain damage. Neurocritical care after severe TBI has therefore been refined to focus not only on secondary brain injury but also on systemic organ damage after excitation of sympathetic nerves following a stress reaction.

## Introduction

When a patient needs neurocritical care after a traumatic brain injury (TBI), several factors must be given focus, such as primary and secondary brain injuries. Primary brain injury is defined by the direct mechanical forces which occur at the time of the traumatic impact to the brain tissue. These forces and the injury they cause to the brain tissue trigger secondary brain injury over time. The impact of secondary brain injury caused by dysautoregulation of brain vessels and blood–brain barrier (BBB) disruption may be magnified by these processes, leading to the development of brain edema, increased intracranial pressure (ICP), and finally, decreased cerebral perfusion pressure (CPP; difference between systemic arterial pressure and ICP; normally ranges approximately between 60 and 70 mmHg). However, these brain injury processes incorporate many clinical factors: depolarization and disturbance of ionic homeostasis [1], neurotransmitter release (e.g., glutamate excitotoxicity) [2], mitochondrial dysfunction [3], neuronal apoptosis [4], lipid degradation [5], and initiation of inflammatory and immune responses [6]. However, the extremely complex nature of these brain injury mechanisms makes it difficult to simply and clearly differentiate between the factors in patients with TBI [7, 8].

The central mechanisms of dysregulation after brain injury may contribute to the development and progression of extracerebral organ dysfunction by promoting systemic inflammation that have the potential for medical complications. Complications such as pneumonia, sepsis, or multiple organ dysfunction syndrome are the leading causes of late morbidity and mortality in many types of brain damage [9–13]. Indeed, the catecholamine surge following systemic insult is directly involved in the regulation of cytokine expression in situations of acute stress [11, 12, 14], producing a worsening clinical condition and, ultimately, a poor outcome [11, 15]. The trauma-induced catecholamine surge affects systemic organs and contributes to organ damage [16]. Neurocritical care after severe TBI has therefore been refined to focus not only on secondary brain injury but also on systemic organ damage after excitation of sympathetic nerves following a stress reaction, including hyperglycemia [17, 18]. This article reviews the pathophysiology with a focus on neurocritical care linked to systemic responses in patients with severe TBI.

## Review

### Regulatory systems of the brain

The normal brain has several mechanisms for regulating pressure and volume. The purpose of these mechanisms is to maintain a continuous cerebral blood flow (CBF) and adequate oxygen supply, despite changes in both systemic arterial pressure (SAP) and cerebral metabolic requirements [19]. The key mechanism is the change in cerebrovascular resistance through vasoconstriction and dilatation that are adjusted using many different mediators [20]. Cerebral pressure reactivity is one of the critical systems in cerebral autoregulation and allows smooth vascular muscle response to changes in SAP. Under physiological conditions, an increase in SAP caused by a compensatory vasoconstriction will lead to increased cerebrovascular resistance, thus keeping the CBF constant [21].

**Fig. 1:**
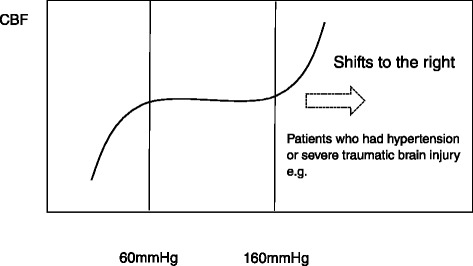
Brain autoregulation (pressure regulation) curve. Cerebral blood flow (CBF) is constant when mean arterial blood pressure (MAP) is kept between 60 and 160 mmHg. As the cerebral vasculature changes to adjust to MAP, vasoconstriction or vasodilatation changes. In patients who had hypertension or severe traumatic brain injury (TBI), the autoregulation curve shifts to the right. Due to the rightward shift (*arrow*), a MAP-dependent CBF reduction (brain ischemia) or increase (hyperemia) occurs even for a small change in blood pressure. Note that the plateau range of CBF is presumably altered after TBI occurs. No clear data are available, however, on how this presumed alteration takes place

Small vessels in the brain thus react to hydrostatic pressure and regulate the vascular tone to maintain a constant CBF between mean arterial pressures (MAP) of 60 and 160 mmHg. When the autoregulation mechanism fails and the BBB is also disrupted, the CBF becomes dependent on SAP, resulting in a critical condition for the injured brain. As can be observed from the pressure regulation curve’s rightward shift in the severely injured brain, accidental changes in SAP can cause severe and linear changes in CBF that lead to harmful and irreversible conditions, such as hypoperfusion (brain ischemia) or hyperperfusion (e.g., hyperemia). These may lead to an irreversible and catastrophic increase in ICP (Fig. 1).

#### Vasodilation and vasoconstriction cascade in cerebral vasculature

**Fig. 2:**
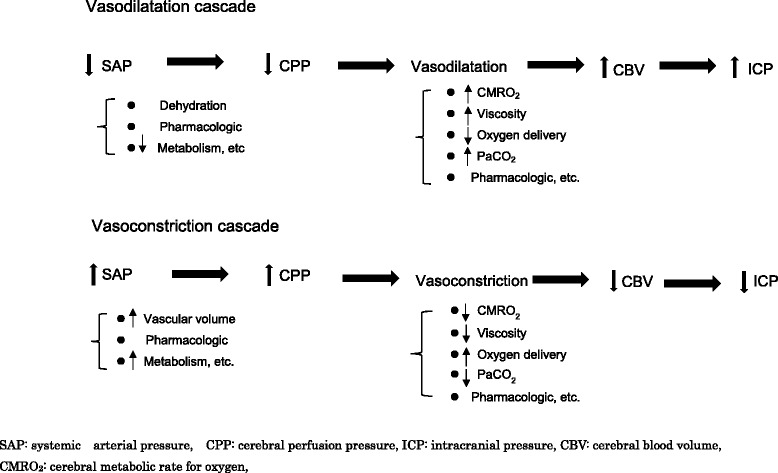
Vasodilation and vasoconstriction cascade in the cerebral vasculature. This cascade model was first described by Rosner in the 1990s (see references 22, 23). A cascade of this type is often trigged by changes in CPP. Any step in the cascade, however, can be triggered as the starting point. There are many triggering factors such as dehydration, vascular volume, systemic metabolism, CMRO_2_, blood viscosity, systemic oxygen delivery, PaCO_2_, or certain pharmacologic agents. *SAP* systemic arterial pressure, *CPP* cerebral perfusion pressure, *ICP* intracranial pressure, *CBV* cerebral blood volume, *CMRO*_*2*_ cerebral metabolic rate for oxygen

With a normally responding cerebral autoregulatory mechanism, the maximum cerebral vasoconstriction response would drive the vascular mechanism to minimize the cerebral blood volume (CBV). Changes in CBV or SAP would lead to vasodilation or constriction of brain vessels as a response in line with the previously reported vasodilation and vasoconstriction cascades [22, 23]. Many factors can initiate the vasodilation and vasoconstriction cascades, including SAP, systemic blood volume, blood viscosity, oxygen delivery/metabolism, hypo/hypercapnia, and pharmacologic agents (Fig. 2).

Cerebral vasodilation could result in decreased SAP, leading to increased CBV and ICP. If the SAP remains low, the CPP will drop further, accelerating the vasodilation cascade until the maximum cerebral vasodilation is attained or SAP can be stabilized. The cascade could also be initiated by hypoxemia, dehydration, or hypercapnia.

Conversely, stimulating a vasoconstriction cascade can sometimes be strategically useful for severe TBI patients. An increase in SAP could stimulate the cerebral vasoconstriction cascade that potentially drives a drop in CBV with a subsequent drop in ICP. If the volume regulatory response is intact (i.e., brain responds normally), an increase in CBV will also accelerate the vasoconstriction cascade, thereby reducing ICP. The vasoconstriction cascade will also contribute to fluid loading, red cell transfusion, viscosity reduction (this means fluid replacement in a clinical setting), or improved oxygen delivery for systemic management in critical care. This cascade could be clinically effective for small volume replacement in low-CPP patients who may be potentially dehydrated. These pressure or volume regulatory cascades may hint at opportunities for the next step in treatment strategies for TBI patients. However, traumatized patients will require careful management since SAP might be maintained due to increased systemic vascular resistance (neurogenic hypertension) after TBI, a condition that often masks a potentially dehydrated condition.

#### Hyperemia after TBI

Hyperemia is associated with elevated CBV and a drop in distal cerebrovascular resistance [24] and frequently observed as “luxury perfusion” following ischemia [25, 26] and/or TBI [24]. Many drivers, such as lactic acid, neuropeptides, and adenosine, generated by vasodilatory metabolites, have been considered to be part of the mechanism for causing a drop in distal cerebrovascular resistance. When pressure autoregulation is intact, a suitable coupling has been observed between a small rise in CBF and metabolism [27, 28]. Alternatively, dysfunctional pressure or volume autoregulation may elicit hyperemia that is associated with intracranial hypertension and an unfavorable outcome [29–31]. If hyperemia combines with BBB disruption, capillary leakage in the dilated vascular bed may cause a brain edema to occur [32]. In the latter process, increased CBF and CBV due to vessel dilation with BBB disruption may lead to aggravated vascular engorgement and brain edema, ultimately leading to “malignant brain swelling,” the development of irreversible intracranial hypertension. If the vasoconstriction cascade is intact and responding normally, hyperventilation therapy has been proposed to reduce PaCO_2_ levels, which might be effective for treating brain swelling.

### Management of patients with TBI

#### Respiratory care

The clinically critical aspect to manage patients with TBI is the minimization of secondary cerebral damage. Hyperventilation therapy for acute-phase patients with severe TBI reduces ICP and improves outcome [33, 34]. However, excessive hyperventilation induces vasoconstriction and subsequent CBF decrease that leads to brain ischemia. Unfortunately, this phenomenon is difficult to detect without any neuromonitoring. A report that discusses the disturbance of cerebral oxygen metabolism balance mentioned the following as causes: (1) hypoxia; (2) hypotension; (3) hypo/hyper PaCO_2_; and (4) anemia. These were extracranial causes comprising 45 % of all causes and were equal to the incidence of dysoxygenation caused by intracranial causes (48 %) that include increased ICP [35]. Therefore, achieving respiratory and hemodynamic stabilization is essential for preventing the progression of secondary brain injury in TBI patients.

**Fig. 3:**
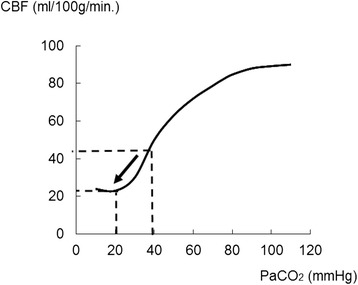
Changes in CBF related to PaCO_2_ level variation. In the case of respiratory acidosis, the effect of PaCO_2_ on the cerebral vasculature can augment cerebral blood flow (CBF). Conversely, CBF would be reduced by vasoconstriction after a drop in PaCO_2_. When PaCO_2_ values fall below 20 mmHg from about 40 mmHg, CBF also drops to half of the basic value (*arrow*)

ICP is significantly influenced by PaCO_2_. Based on the cerebrovascular CO_2_ reactiveness, a brain blood vessel dilatation caused by a rise in PaCO_2_ may induce an ICP increase and contribute to an increase in CBV (brain swelling), likely resulting in a poor outcome for patients with severe TBI. In contrast, when PaCO_2_ drops, the brain blood vessel shrinks, leading to a decrease in CBV and ultimately to a drop in ICP. When hypercapnia develops after a TBI, such as an airway obstruction or respiratory insult, hyperventilation therapy may be effective for decreasing the ICP when the patient’s CO_2_ reactivity in the cerebral vasculatures is preserved. As this specific condition often occurs in a pre-hospital setting or an emergency room, paramedics or physicians must carefully observe the patients’ respiratory conditions. However, if the PaCO_2_ value falls to 20 mmHg or less from about 40 mmHg, the CBF might fall to half of what it was at 40 mmHg (Fig. 3, arrow), accelerating brain ischemia and causing increased ICP [36–38]. Therefore, excessive hyperventilation therapy should be avoided after TBI, especially within 24 h of the injury [39, 40].

Positive end-expiratory pressure (PEEP) is one key factor for maintaining oxygenation. Application of PEEP may decrease the cerebral venous drainage by raising the intrathoracic pressure and thereby increase the CBV and ICP. PEEP may also increase ICP when the baseline ICP is lower than PEEP, but it has less effect on cerebral perfusion when ICP is above the highest applied PEEP [41]. Hence, mild to moderate PEEP could be effective in preventing ventilator-associated lung injury and increased ICP [42]. The lowest level of PEEP that maintains adequate oxygenation and prevents end-expiratory collapse, usually 5 to 8 cm H_2_O, is recommended. Higher PEEP, up to 15 cm H_2_O, may be used in cases of refractory hypoxemia [43] in spite of its controversial effects on ICP after TBI.

#### Hemodynamic care

In patients with severe TBI and hypotension, acute brain swelling is often observed after SAP elevation efforts using vasopressors or excessive fluid resuscitation. Elevating SAP with large-volume fluid resuscitation or blood transfusion is one critical approach for patients with severe TBI. Although these approaches aggravate brain swelling and increase ICP, identifying dysautoregulation or/and BBB disruption is very difficult. BBB disruption also leads to the formation of brain edema. Brain edema after TBI can be of cytotoxic or vasogenic origin [44, 45] or may be caused by capillary leakage, a risk in TBI that also leads to brain edema. Under these conditions, a high CPP may be harmful even in the case of a relatively intact autoregulation response [45].

Hemodynamic management for patients with TBI has been discussed at length [46, 47]. CPP management is one of the critical strategies that focuses on pressure response [48]. During CPP management with norepinephrine for increasing MAP, the risk of hyperemia could be reduced if pressure autoregulation is preserved [49]. While there is no standard regimen for patients in hemorrhagic shock with TBI complications, the goal of fluid resuscitation for these patients is 60 mmHg of CPP or greater, or if CPP of patients with severe TBI is measurable, the target systolic SAP is 90–100 mmHg instead of achieving normal SAP.

Hypotension is frequently observed after TBI [50, 51] and might affect the outcome. An increase in endogenous catecholamines (sympathetic-excited catecholamine surge) causes vasoconstriction of peripheral vessels that elevates SAP (neurogenic hypertension) after TBI. As a result, SAP is maintained even if the hypovolemia exists. Mannitol has historically been used for patients with elevated ICP as an osmotic diuretic [52, 53]. However, excessive intravascular dehydration by inappropriate mannitol use leads to dehydration and degrades hemodynamics to an unstable state, whereupon unanticipated hypotension occurs [51]. If intracranial hypertension is also suddenly relieved by surgical decompression craniotomy, the sympathetic response is eliminated, which may elicit systemic hypotension caused by reduced vascular resistance (vasodilation) [45]. Under conditions where the BBB is disrupted or/and cerebrovascular permeability increases after TBI, brain swelling may occur when massive fluid resuscitation and blood transfusion is administered to treat hypotension [50, 51]. To prevent catastrophic hypotension and brain swelling after TBI during critical care or surgery, the routine use of mannitol administration and intravascular dehydration should be avoided. Normovolemia must be maintained during critical care.

#### Monitoring CBF and metabolism balance

Jugular bulb oxygen saturation (SjO_2_) provides information on global cerebral oxygen delivery and metabolism, which is used for detecting cerebral hypoperfusion, hyperperfusion, or secondary ischemic brain injury [54–56].

**Fig. 4:**
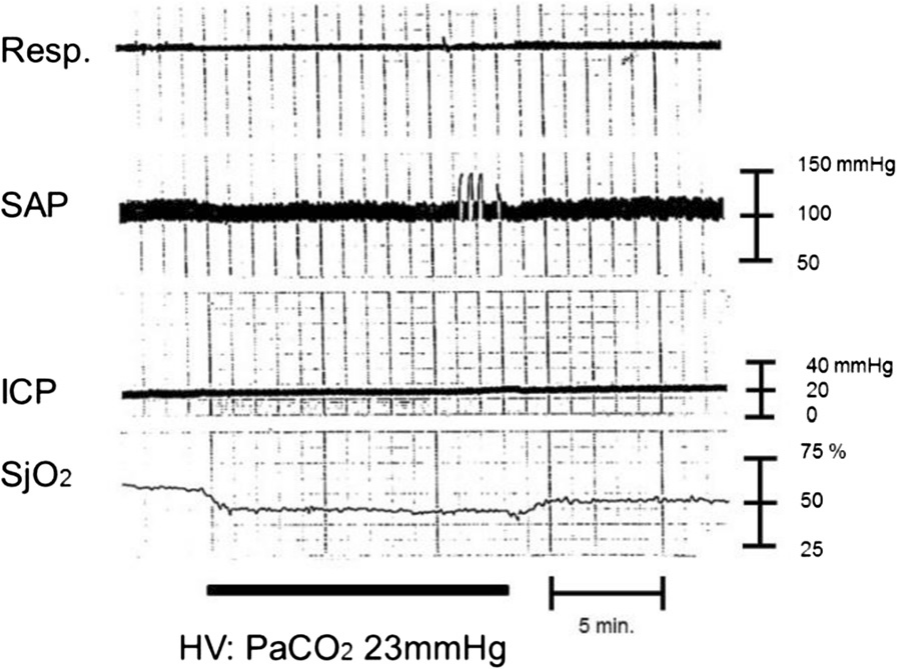
Brain ischemia after hyperventilation. A female in her 40s with traumatic brain injury was transferred to the hospital by ambulance. Brain CT scan revealed acute subdural hematoma. Surgical interventions were performed, and the patient’s ICP and SjO_2_ were monitored. The SjO_2_ value drops after hyperventilation. This phenomenon can be explained by the vasoconstriction effect from reduced PaCO_2_. Cerebral perfusion pressure changes might not have any remarkable effect because SAP and ICP values have been constant. Clinically, physicians would not be able to detect brain ischemia only from vital signs in this case without monitoring for brain oxygenation, such as SjO_2_ monitoring. The ICP will stay constant even if there are changes in the intracranial volume (e.g., the change in the volume of the vascular bed during the space compensatory phase). While the ICP will spread to the CSF space or any similar space until the compensatory effect is lost, no remarkable changes in the ICP are seen during the space compensatory phase. As a consequence, hyperventilation therapy for ICP control will not be effective in this phase. It may even cause harm via the decrease in CBF induced by excess vasoconstriction. *Resp.* respiration, *SAP* systemic arterial pressure, *ICP* intracranial pressure, *SjO*_*2*_ jugular bulb oxygen saturation, *HV* hyperventilation. Data were obtained from brain injury patient monitored at our hospital in the 1990s

**Fig. 5:**
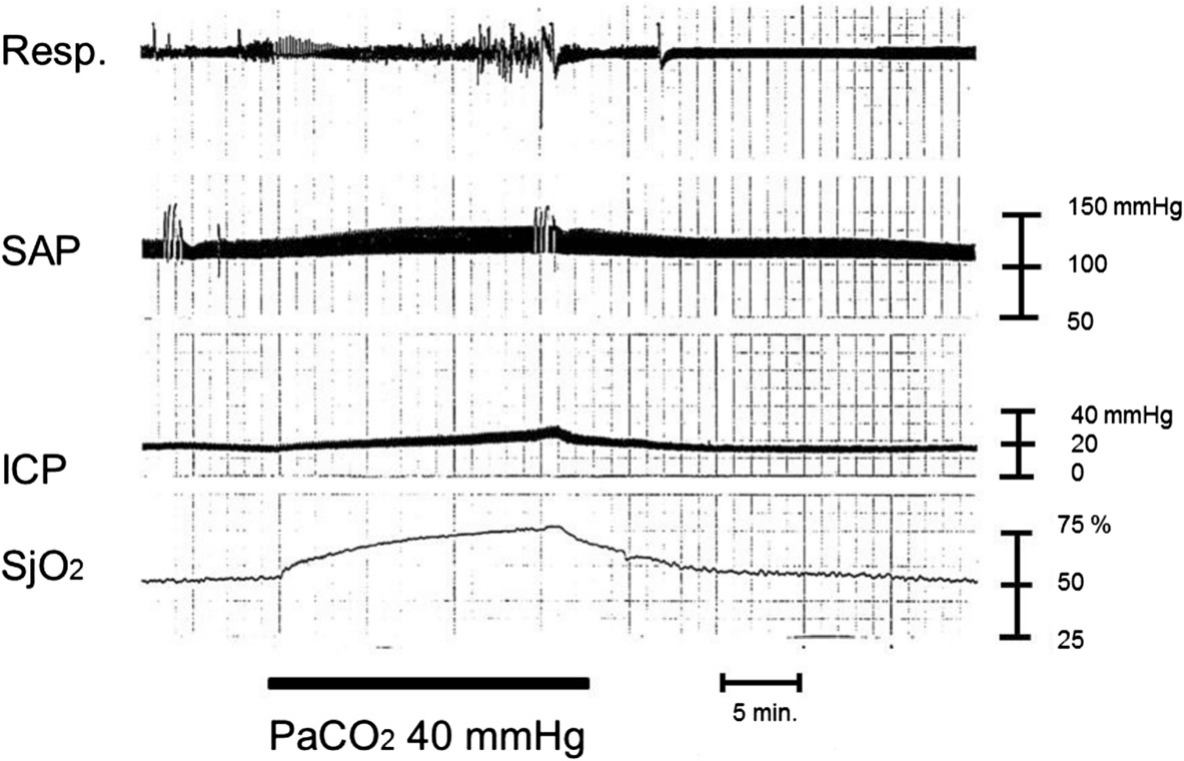
Effect on cerebral blood flow caused by augmentation of PaCO_2_. A male in his 30s suffered a traffic accident. Initial CT scan demonstrated acute subdural hematoma. Increased PaCO_2_ could stimulate the vasodilation cascade in the brain. As a result of an increase in PaCO_2_, the brain vasculature goes through vasodilation, with a subsequent increase in cerebral blood flow (and cerebral blood volume), leading to increased ICP. Physicians would be able to detect this from increased SjO_2_ in the clinical setting. *Resp.* respiration, *SAP* systemic arterial pressure, *ICP* intracranial pressure, *SjO*_*2*_ jugular bulb oxygen saturation, *CPP* cerebral perfusion pressure. Data were obtained from brain injury patient monitored at our hospital in the 1990s

The normal SjO_2_ level is approximately 60 %. SjO_2_ values under 50 % is considered to be cerebrally ischemic when accompanied by low CBF or/and CPP [54]. High SjO_2_ values may reflect hyperemia (higher CBF and dilatation of blood vessels; increased CBV) or severe metabolic depression due to severe brain damage. Continuous SjO_2_ monitoring is effective for detecting cerebral ischemia after TBI [57]. SjO_2_ monitoring is most commonly used for severely brain-injured patients to detect post-injury brain ischemia and to monitor the efficacy of mannitol injection or hyperventilation therapy. If hyperventilation becomes excessive, cerebral vasoconstriction will occur and ultimately lead to further aggravation of cerebral perfusion of the already injured brain (reduced CPP that leads to brain ischemia). Figure 4 indicates the relationship between hyperventilation and sequential changes in SjO_2_. Excessive hyperventilation can cause a drop in PaCO_2_, leading to vasoconstriction, and then result in brain ischemia, based on the SjO_2_ level (the SjO_2_ value drops during excess hyperventilation as demonstrated in Fig. 4). Conversely, heightened PaCO_2_ values lead to higher SjO_2_ levels (Fig. 5). This phenomenon is caused by the effect of greater CBV on vasodilation (vascular bed enhancement).

**Fig. 6:**
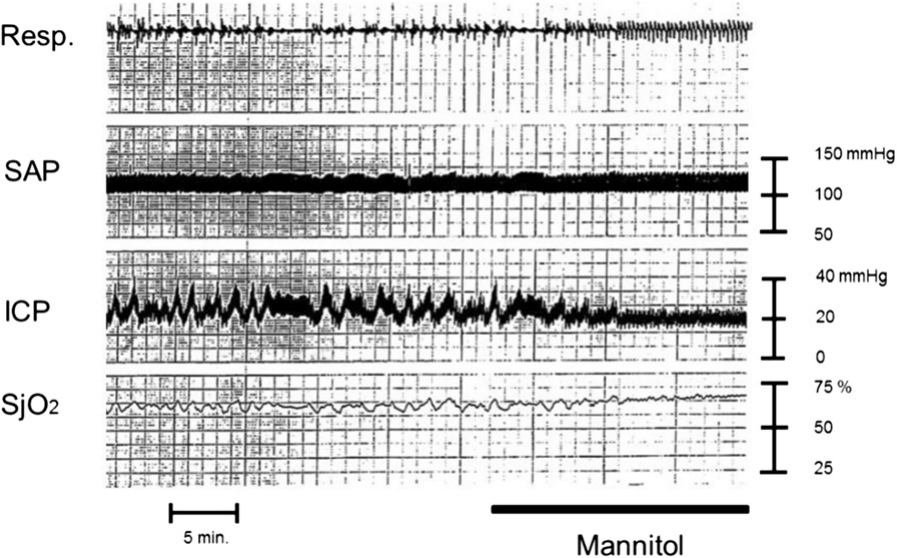
Effect of mannitol administration on patient with intracranial hypertension. A male in his 60s suffered traumatic brain injury. Brain CT scan demonstrated cerebral contusion. Mannitol administration is a potentially effective volume replacement method in the early phase and can stimulate the vasoconstriction cascade. SjO_2_ values gradually increase after mannitol administration. This phenomenon is likely caused by the volume expansion effect of mannitol, which could stimulate the vasoconstriction cascade leading to decreased CBV. Mannitol will then work as a hyperosmotic diuretic agent at the late phase resulting in decreased ICP and increased CPP. *Resp.* respiration, *SAP* systemic arterial pressure, *ICP* intracranial pressure, *SjO*_*2*_ jugular bulb oxygen saturation, *CBV* cerebral blood volume, *CPP* cerebral perfusion pressure, *Mannitol* mannitol administration. Data were obtained from brain injury patient monitored at our hospital in the 1990s

The vasodilatation of brain vessels is triggered by a drop in CPP with a subsequent CBV increase [22]. The drop in CPP is often associated with a decrease in SAP. CPP can be boosted by infusing fluids or by administering mannitol (as a volume expander) or vasopressors, with a subsequent vasoconstriction of brain blood vessels [58] (Fig. 6). Finally, ICP can be lowered as a result of reduced CBV after vasoconstriction [22, 58]. Above the upper autoregulated limit, hyperperfusion may be a risk for hyperemia. Conversely, a drop in SAP at the lower limit for autoregulation response may reduce CPP and cause brain ischemia. Increased ICP levels may lead to further reductions in CPP.

#### Catecholamine surge after severe brain injury

Catecholamine surge is a well-known phenomenon that is observed after subarachnoid hemorrhage [59], sepsis [10], or TBI [13], where such elevated levels appear to influence the immune system during stress. In particular, the results from stressed subjects have highlighted a close relationship between the cytokine network, systemic inflammatory response syndrome, and the immune response [60, 61], while pro-inflammatory cytokines (e.g., interleukin (IL)-1) can enhance the sympathetic nerve activity [62, 63]. Remarkably, in vitro studies have demonstrated that epinephrine or norepinephrine upregulated the endotoxin-induced release of anti-inflammatory cytokine IL-10 from human peripheral blood mononuclear cells (macrophages/monocytes), whereas tumor necrosis factor-alpha production was downregulated [64–66]. Indeed, the catecholamine surge could suppress mononuclear cell functions, which are upregulated by immunostimulatory cytokines. Such functional suppression is also observed in patients with sepsis [67, 68], burns [69], and trauma [12, 70]. This phenomenon may play an important role in early immunosuppression in patients suffering an acute stressful event.

#### Brain injury and hyperglycemia

Hyperglycemia is also a well-known phenomenon that is observed after stressful events such as severe brain damage. The adverse effects of hyperglycemia on ischemic brain injury have been well established in both the clinical and experimental settings. While the clinical evidence indicates that high blood glucose levels following TBI are linked to a greater severity of injury and poor neurological outcome [17, 18], the role of blood glucose in the secondary mechanisms of neuronal damage after TBI has not yet been clarified. Data from brain ischemia models suggest that hyperglycemia has a deleterious effect, probably due to enhanced lactic acidosis. Previous studies have demonstrated that hyperglycemia causes a variety of pathological changes in the small vessels, arteries, and peripheral nerves. Vascular endothelial cells are a significant target of hyperglycemic damage [71], but the mechanisms underlying such damage to the cerebral microvasculature are not fully understood. Several authors have reported that hyperglycemia leads to endothelial dysfunction [72] and cerebrovascular changes both during ischemia and reperfusion [73]. Recently, nuclear factor-kappa B activation has been identified as an early event brought about by elevations in glucose, which may elicit multiple pathways contributing to the initiation of hyperglycemia- or diabetes-induced endothelial cell injury. It also plays a pivotal role in early gene responses following hyperglycemia by promoting messenger RNA synthesis for various cell-adhesion molecules, inducible nitric oxide synthase, and cytokines or chemokines [74]. These inflammatory events are believed to contribute to the observed outcomes through secondary injury mechanisms [75, 76]. Additionally, acute inflammatory responses lead to the activation of infiltration and accumulation of polymorphonuclear leukocytes [77].

It has been proposed that hyperglycemia may contribute to endothelial cell damage in brain ischemia models [78] and TBI [79]. We have yet to gain a clear understanding, however, of the exact mechanisms by which the neutrophil transmigration across the BBB is enhanced under the hyperglycemic condition following TBI. Experimental studies have shown that a hyperglycemic condition activates the intracellular signal transduction [80, 81] and production of interleukin (IL)-8 [82]. The presence of tumor necrotic factor (TNF) in a high-glucose condition could enhance the production of IL-8 from endothelial cells [82]. We speculate that the hyperglycemic environment and the severe trauma associated with elevated TNF might work in combination to promote IL-8 production by vascular endothelial cells and foster neutrophil accumulation at the injury site. This, together with the hyperglycemia after TBI, may aggravate the endothelial cell damage and enhance the inflammatory process, leading to neutrophil infiltration into the injured brain.

In the clinical setting, however, a frequent post-hospitalization event in patients with severe brain injury is a rapid and large increase in blood glucose concentration that occurs in various situations. Several questions also remain as to when patients with severe brain injury should be started on glucose-containing IV fluids for maintenance alimentation, since acute hyperglycemia may influence the neurological outcome. However, the potential for acute hyperglycemia on its own to cause inflammation in brain tissue following an acute critical illness, including neutrophil accumulation, has not been investigated much.

## Conclusions

Severe brain injury involves impaired autoregulation and responses in the injured brain through many mechanisms that lead to secondary brain injuries. Arterial hypotension, hypertension, or excess hyperventilation intended to reduce ICP in patients with damaged autoregulation response also lead to secondary brain injury and critical brain conditions after TBI that are associated with a poor outcome. The central dysregulation mechanisms after brain injury could contribute to the development and progression of extracerebral organ dysfunction by promoting systemic inflammation that may cause medical complications. Neurocritical care after severe TBI has therefore been refined to focus not only on secondary brain injury but also on systemic organ damage after excitation of sympathetic nerves following stress reactions.

## Key points of the “pathophysiology for neurocritical care” in traumatic brain injury

- Cerebral autoregulation is one of the important pressure reactivity systems in the brain. The small vessels in the brain react to hydrostatic pressure and regulate the vascular tone to maintain a constant cerebral blood flow between the mean arterial pressures of 60 and 160 mmHg. As the pressure regulation curve shifts rightward in the severely injured brain, accidental changes in systemic arterial pressure can cause severe and linear changes in cerebral blood flow that lead to harmful and irreversible conditions such as hypoperfusion (brain ischemia) or hyperperfusion (e.g., hyperemia).
- Changes in cerebral blood volume or systemic arterial pressure lead to vasodilation or constriction of brain vessels. Cerebral vasodilation may result in decreased systemic arterial pressure leading to increased cerebral blood volume and intracranial pressure. The response could also be initiated by hypoxemia, dehydration, or hypocapnia due to hyperventilation therapy.
- A drop in cerebral perfusion pressure triggers vasodilation of the cerebral blood vessels and subsequent increase of the cerebral blood volume. The drop in cerebral perfusion pressure is often associated with a decrease in systemic arterial pressure. Above the upper autoregulated limit, hyperperfusion may heighten the risk of hyperemia. Conversely, a drop in systemic arterial pressure at the lower limit for autoregulation response may reduce cerebral perfusion pressure and cause brain ischemia.
- Excessive hyperventilation induces vasoconstriction and a subsequent reduction of cerebral blood flow that leads to brain ischemia. Based on the cerebrovascular CO_2_ reactivity, a brain blood vessel dilatation caused by a rise in PaCO_2_ may increase the intracranial pressure and contribute to an increase in the cerebral blood volume (brain swelling). The outcome is likely to be poor for patients with severe traumatic brain injury when this occurs. When PaCO_2_ drops, on the other hand, the brain blood vessel shrinks, leading to a decrease in cerebral blood volume and ultimately a drop in intracranial pressure.
- An increase in endogenous catecholamines (sympathetic-excited catecholamine surge) causes vasoconstriction of peripheral vessels that elevates systemic arterial pressure (neurogenic hypertension) after traumatic brain injury. As a result, systemic arterial pressure is maintained even if the hypovolemia exists. Mannitol has historically been used for patients with elevated intracranial pressure as an osmotic diuretic. When used inappropriately, however, mannitol induces excessive intravascular dehydration. The resulting dehydration and degraded hemodynamics lead to an unstable state and unanticipated hypotension. To prevent unexpected catastrophic hypotension after TBI, the routine use of mannitol and intravascular dehydration should be avoided.
- Hyperglycemia also frequently develops after severe brain damage or similarly stressful events. High blood glucose levels following traumatic brain injury are apparently associated with more severe injuries and poor neurological outcomes. Little is still known, however, about the action of blood glucose in the secondary mechanisms of neuronal damage after traumatic brain injury. The best time to commence glucose-containing IV fluids for maintenance alimentation is also uncertain, since acute hyperglycemia may alter the neurological outcome. It remains to be determined, however, if hyperglycemia alone can readily cause brain tissue inflammation after an acute critical illness involving neutrophil accumulation.

## Abbreviations

BBB: blood–brain barrier
CBF: cerebral blood flow
CBV: cerebral blood volume
CPP: cerebral perfusion pressure
ICP: intracranial pressure
MAP: mean arterial pressure
SAP: systemic arterial pressure
SjO_2_: jugular bulb oxygen saturation
TBI: traumatic brain injury
